# Reno-Protective Effect of GLP-1 Receptor Agonists in Type1 Diabetes: Dual Action on TRPC6 and NADPH Oxidases

**DOI:** 10.3390/biomedicines9101360

**Published:** 2021-09-30

**Authors:** Natalie Youssef, Mohamed Noureldein, Rachel Njeim, Hilda E. Ghadieh, Frederic Harb, Sami T. Azar, Nassim Fares, Assaad A. Eid

**Affiliations:** 1 Department of Anatomy, Cell Biology and Physiological Sciences, Faculty of Medicine, American University of Beirut, Bliss Street, 11-0236, Riad El-Solh, Beirut 1107-2020, Lebanon; nsy05@mail.aub.edu (N.Y.); mhn13@mail.aub.edu (M.N.); rmn32@mail.aub.edu (R.N.); hg36@aub.edu.lb (H.E.G.); 2 American University of Beirut (AUB) Diabetes, American University of Beirut, Bliss Street, 11-0236, Riad El-Solh, Beirut 1107-2020, Lebanon; sazar@aub.edu.lb; 3 Department of Life and Earth Sciences, Faculty of Sciences, Lebanese University, Fanar, Jdeidat P.O. Box 90656, Lebanon; frederic.harb@ul.edu.lb; 4 Department of Internal Medicine, Faculty of Medicine, American University of Beirut, Bliss Street, 11-0236, Riad El-Solh, Beirut 1107-2020, Lebanon; 5 Laboratory of Physiology and Physiopathology, Faculty of Medicine, Saint Joseph University of Beirut, Damas Street, 11-5076, Riad El-Solh, Beirut 1107-2180, Lebanon

**Keywords:** diabetic kidney disease, DUOX1/2, TRPC6, GLP-1, liraglutide

## Abstract

Diabetic kidney disease (DKD), a serious diabetic complication, results in podocyte loss and proteinuria through NADPH oxidases (NOX)-mediated ROS production. DUOX1 and 2 are NOX enzymes that require calcium for their activation which enters renal cells through the pivotal TRPC channels. Hypoglycemic drugs such as liraglutide can interfere with this deleterious mechanism imparting reno-protection. Herein, we aim to investigate the reno-protective effect of GLP1 receptor agonist (GLP1-RA), via its effect on TRPC6 and NADPH oxidases. To achieve our aim, control or STZ-induced T1DM *Sprague–Dawley* rats were used. Rats were treated with liraglutide, metformin, or their combination. Functional, histological, and molecular parameters of the kidneys were assessed. Our results show that treatment with liraglutide, metformin or their combination ameliorates DKD by rectifying renal function tests and protecting against fibrosis paralleled by restored mRNA levels of nephrin, DUOX1 and 2, and reduced ROS production. Treatment with liraglutide reduces TRPC6 expression, while metformin treatment shows no effect. Furthermore, TRPC6 was found to be directly interacting with nephrin, and indirectly interacting with DUOX1, DUOX2 and GLP1-R. Our findings suggest that treatment with liraglutide may prevent the progression of diabetic nephropathy by modulating the crosstalk between TRPC6 and NADPH oxidases.

## 1. Introduction

Diabetes mellitus is a leading cause of mortality worldwide. It is associated with deleterious complications that affect the eyes (retinopathy), nerves (neuropathy), kidneys (nephropathy), heart, and blood vessels causing dysfunction of the affected organs. Diabetes imposes a major economic burden on the global healthcare system [1].

A hallmark of diabetic nephropathy, also termed diabetic kidney disease (DKD), is the disruption of the glomerular filtration barrier (GFB) and thickening of the glomerular basement membrane (GBM) [2]. A central actor for these pathological mechanisms is podocytes; their loss has a fatal impact on the GFB, as podocyte loss cannot be compensated, and is irreversible [3]. The actin cytoskeleton and associated proteins such as nephrin are essential for podocytes to sustain the integrity of the slit diaphragm, and thereby maintain adequate filtration [4].

Considerable efforts are continuously made to elucidate the mechanisms underlying the development of DKD. It has been shown that increased reactive oxygen species (ROS) production and impairment of nephrin expression play a major role in the development of DKD [5]. Among the different sources of ROS production, nicotinamide adenine dinucleotide phosphate (NADPH) oxidases are considered to be the major driver, and co-player with transient receptor potential canonical (TRPC) 6 in mediating podocyte injury [6]. The NADPH oxidase (NOX) family consists of seven transmembrane protein members (NOX 1 to 5 and dual oxidases (DUOX) 1 and 2) [7]. DUOXs are unique in their calcium sensitivity. Therefore, their interaction with calcium influx in podocytes is of high interest to investigate.

Calcium signaling is of key importance in renal cells function, and the disruption of its handling may lead to pathological manifestations such as those observed in diabetic nephropathy. The calcium permeable TRPC channels control calcium influx in podocytes and play an essential role in diabetic nephropathy [8,9]. All of the seven members of TRPC channels are expressed in podocytes except TRPC2 and TRPC7 [10]. Evidence has shown that calcium influx into the podocytes is governed by TRPC3, TRPC5 and TRPC6 [11], with TRPC6 upregulation suggested to play a key role in podocyte loss and detachment. In patients with diabetes, hyperglycemia and hyperinsulinemia may result in induction of TRPC6 expression and subsequent calcium influx into the podocytes [12]. Remarkably, these factors are also associated with increased ROS production [13]. Moreover, TRPC6 over-expression has been linked with oxidative stress and podocyte injury [14]. Thus, studying the interaction of NADPH oxidases with TRPC6 in podocytes is of great value.

Given the impact of DKD on the survival of patients with diabetes, several hypoglycemic agents were investigated for their reno-protective effects. Of these hypoglycemic agents, liraglutide, a glucagon-like peptide -1 (GLP-1) receptor agonist appears to be the most promising.

Glucagon-like peptide-1 receptor agonists (GLP1-RA) are a wide class of hypoglycemic agents used in the treatment of type 2 diabetes. Beyond glycemic control, recent data from clinical trials have demonstrated the reno-protective effects of GLP1-RA, with a special focus on liraglutide [15]. Substantial efforts have been made to understand the involvement of GLP-1 in DKD and to establish the mechanisms behind the reno-protective effects of GLP-1RA. Direct and indirect mechanisms for GLP1-RA reno-specific actions have been proposed. The direct mechanism includes the classic signaling pathway involving cAMP/protein kinase A (PKA), natriuresis, renal perfusion modulation and ischemia prevention [15]. The indirect mechanisms include lowering glucose levels, improving insulin sensitivity, reducing blood pressure and weight loss [15]. Despite all the efforts exerted to study GLP1-RA reno-protective role, limited studies have investigated the effect of these reno-protective agents in type 1 diabetes.

On the other hand, metformin is a commonly prescribed oral hypoglycemic agent for patients with type 2 diabetes and has been described to confer renal and cardiovascular protective effect in this category of patient with diabetes. However, the data of metformin use as a reno-protective agent in clinical studies of type 1 diabetes remain inconclusive [16,17]. Our group and others have previously described metformin as an activator of 5′-adenosine monophosphate-activated kinase (AMPK) [18,19]. Besides, we have previously shown that by activating AMPK using metformin in the type 1 diabetic mice, the observed reno-protection was associated with a decrease in NADPH oxidase-mediated ROS production [18]. Yet, in the latter study as well as in other studies [20], the crosstalk of metformin with calcium channels specifically TRPC6 was not delineated.

In this study, we show that the absence of proper GLP-1R activity predisposes the hyper-activation of DUOX1 and DUOX 2 isoforms of the NADPH oxidases enzymes in the diabetic milieu, leading to increased ROS production. The produced ROS activates TRPC6 channels increasing calcium influx, which eventually leads to diabetic kidney injury. Given the above-proposed mechanism, the present work shows that liraglutide, a GLP1-RA, confers reno-protection by correcting the alteration observed in the NAPDH and TRPC6 signaling axis.

## 2. Materials and Methods

### 2.1. Animal Studies

Animal procedures were approved by the institutional animal care and use committee (IACUC) of the American University of Beirut, (IACUC protocol number 19-12-565, approval date: 11 December 2019) according to the guidelines of the National Institutes of Health (NIH). Male *Sprague–Dawley* rats were allocated into five different groups. A control group (C) (*n* = 4) was treated with citrate buffer alone (0.01 M, pH = 4.5), while the other rats were rendered type 1 diabetic through a single intravenous (IV) injection of 55 mg/kg streptozotocin (STZ) (Sigma-Aldrich, Darmstadt, Germany) dissolved in citrate buffer (pH 4.5). Rats were considered diabetic when their fasting blood glucose level was more than or equal to 250 mg/dL. After the onset of type 1 diabetes, diabetic rats were assigned into four treatment groups (*n* = 4 per group). The control and diabetic group were treated with saline (vehicle) and served as the disease reference for the other treatment groups. One group of diabetic rats received intraperitoneal (i.p.) injection 150 mg/kg body weight of metformin once daily (DM). Another group received 0.3 mg/kg body weight of liraglutide, twice daily by subcutaneous (s.c.) injections (DL). The last group received a combination of both i.p. metformin (150 mg/kg) once daily and s.c. liraglutide (0.3 mg/kg) twice daily (DML). Drugs were administered for a duration of 8 weeks. The doses of metformin or liraglutide used in our study were calculated based on the maximum doses given and tolerated by patients with diabetes in clinical settings [21,22]. Besides, the treatment timeline was performed based on previous work conducted by our team [23,24]. No loss of weight or death were observed among the animals after the treatment protocol. Body weight and blood glucose were measured weekly. At the 8th week of treatment, rats were placed in metabolic cages for 24 h for urine collection, and the 24 h urine volume was recorded for each rat. Urine was centrifuged at 1000 rpm for 5 min at 4 °C and the supernatant was collected to be analyzed for proteinuria.

### 2.2. Sacrifice and Organ Harvesting

After the completion of the treatment period, all animals were sacrificed. Blood was collected and centrifuged at 3000 rpm for 15 min at 4 °C. Plasma was gathered and stored at −20 °C for further analysis. Kidneys were harvested and weighed, their capsule removed, and sections were collected in 4% p-formaldehyde for histological analysis, while the rest of the kidney tissue was frozen and properly stored at −80 °C for protein and mRNA extraction.

### 2.3. Blood–Urine Biochemical Analysis

Plasma was assessed for blood urea nitrogen (BUN) and serum creatinine (SCr) using specialized kits (Caymen Chemical, Ann Arbor, MI, USA) according to manufacturer’s instructions.

To measure HbA1c%, 50 μL of whole blood was used. HbA1c levels were determined with a Rat HbA1c Kit (Crystal Chem, Elk Grove Village, IL, USA), according to the manufacturer’s instructions and normalized against total hemoglobin to get HbA1c %.

Additionally, proteinuria was determined using the Bradford assay on an aliquot of 24 h collected urine as previously described [25].

### 2.4. mRNA Extraction and cDNA Synthesis

Extraction of mRNA from kidney tissue was performed using the TRIzol method as previously described [26]. RNA quantification was performed using nanodrop (Denovix, Wilmington, DE, USA). The extracted mRNA was then converted to cDNA. The Quantitect Reverse Transcription Kit (Qiagen, Hilden, Germany) was used according to the manufacturer instructions.

### 2.5. Real-Time PCR

Two µL of cDNA template was used to which 5 µL of SYBR green master mix (which includes Taq polymerase enzyme, EVA green dye, MgCl2, reaction buffer and oligonucleotide) was added. Thirty nL (0.3 µL) of the specific forward and reverse primers of the gene of interest were added to each reaction and the volume was completed with RNase-free water to reach 10 µL. Reactions were run on the RT-PCR Bio-Rad CFX384 (Bio-Rad, Hercules, CA, USA) platform. Primers used are included in (Supplementary Table S1).

### 2.6. Periodic Acid-Schiff (PAS) and Masson’s Trichrome (MT) Staining

The formalin-fixed kidney tissues were embedded in paraffin blocks and cut into 5 μm sections. PAS stain was used to detect any glomeular or tubular fibrosis, while MT stain was used to detect collagen deposition. Quantification of various stains was done using NIH ImageJ software (Wayne Rasband, Kensington, MD, USA) [27].

### 2.7. AMPK Activity Assay

AMPK activity was measured using the AMPK KinEASETM FP fluorescein green assay fluorescence polarization assay (MerckMillipore, Darmstadt, Germany) according to the manufacturer’s protocol [28].

### 2.8. Immunohistochemistry for TRPC6

Immunohistochemistry was performed using Novolink Polymer Detection System Kit (RE7150-K, Leica Biosystems, Buffalo Grove, IL, USA) according to manufacturer’s instructions. TRPC6 was detected using rabbit polyclonal TRPC6 antibody (dilution 1:25), Sigma SAB4300572 (Sigma-Aldrich, Darmstadt, Germany).

### 2.9. Western Blot

Proteins were extracted using 200 μL Radio-immune Precipitating Assay (RIPA) buffer. Samples were then centrifuged at 13,200 rpm for 30 min at 4 °C. Total proteins were quantified using the Lowry protein assay [29]. Western Blot was performed, and the membranes were incubated overnight with rabbit polyclonal TRPC6 (dilution 1:250), Sigma SAB4300572 (Sigma-Aldrich, Darmstadt, Germany), goat polyclonal anti-GLP-1R (dilution 1:350), abcam ab186051 (abcam, Waltham, MA, USA), mouse monoclonal anti-HSC-70 (dilution 1:1000), Santa Cruz sc-7298(Santa Cruz, Dallas, TX, USA). Quantification of protein bands was done using the NIH ImageJ software (Wayne Rasband, Kensington, MD, USA) [30].

### 2.10. Detection of Intracellular Superoxide in Kidney Cortex Using HPLC

Cellular superoxide production was evaluated by HPLC analysis of dihydroethidium (DHE)-derived oxidation products. HPLC-based assay permits the separation of the superoxide-specific 2-hydroxyethidium (EOH) from the non-specific ethidium [31].

### 2.11. NADPH Oxidase Activity Analysis

NADPH oxidase activity was analyzed and calculated in kidney cortex homogenates via the lucigenin-enhanced chemiluminescence method [31].

### 2.12. Protein Interactions and Pathway Analysis

The GeneMANIA database (University of Toronto, TO, Canada) was queried for detection of protein interactions between TRPC6 and other proteins in humans. The GeneMANIA database is a precomputed global resource for the exploration and analysis of associations between proteins through investigation of genomic associations [32]. The enriched pathways associated with these interactions were additionally extracted from the aforementioned database using the Gene Ontology (GO) functional enrichment function.

### 2.13. Statistical Analysis

Statistical analysis was performed using the GraphPad prism software (GraphPad Inc., San Diego, CA, USA). All data are expressed as mean ± SEM (standard error of mean). The analysis of variances (ANOVA) test was used to compare different groups (*n* ≥ 4/group). *p*-value ≤ 0.05 was considered significant. The (*) symbol is used to denote significance versus (vs.) the control group while the (#) symbol was used to denote significance vs. the diabetic non-treated group.

## 3. Results

### 3.1. Metabolic Parameters

Type 1 diabetes mellitus (T1DM) male *Sprague–Dawley* rats show a significant increase in blood glucose levels and HBA1c % (600 ± 0.1 * mg/dL vs. 108.8 ± 4.5 mg/dL and 9.9 ± 1.03% * vs. 5.3 ± 0.05% respectively). These parameters were not affected by the treatments with liraglutide (590.8 ± 9.3 mg/dL vs. 600 ± 00.1 mg/dL and 10.2 ± 1.08% vs. 9.9 ± 1.03%), metformin (538 ± 35.9 mg/dL vs. 600 ± 00.1 mg/dL and 9.5 ± 0.97% vs. 9.9 ± 1.03%) or their combination (528 ± 72.0 mg/dL vs. 600 ± 00.1 mg/dL and 9.8 ± 0.92% vs. 9.9 ± 1.03%).

### 3.2. Liraglutide, Metformin or Their Combination Reverse Renal Dysfunction Observed in T1DM Rats

**Figure 1:**
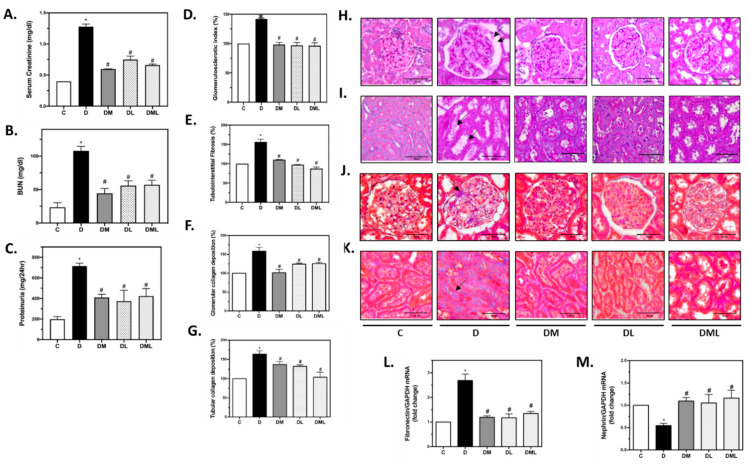
Effect of the GLP1-RA, liraglutide, the AMPK activator, metformin or their combination on kidney injury. (**A**,B) Bar plots representing SCr and BUN concentrations respectively. (**C**) Bar plot reflecting the quantification of proteinuria. (**D**,**E**) Bar plots representing the glomerulosclerotic and tubulointerstitial fibrosis index (%) respectively. (**H**,**I**) Representative microphotographies of glomerular and tubular PAS stain respectively with black arrows pointing to the injury (magnification 40×, scale bar 50 µm). (**F**,**G**) Bar plots representing the quantification of the glomerular and tubular collagen deposition (%). (**J**,**K**) Representative microphotographies of glomerular and tubular MT stain with black arrows pointing to the collagen deposition (Magnification 40×, scale bar 50 µm). (**L**,**M**) Bar plots representing the differential expression of fibronectin and nephrin mRNA expression normalized against GAPDH expression respectively. GAPDH: glyceraldehyde 3-phosphate dehydrogenase. All data are expressed as mean ± SEM. * *p* ≤ 0.05 diabetic vs. control groups. # *p* ≤ 0.05 treated vs. diabetic groups.

To monitor kidney function, SCr, BUN levels as well as protein excretion were measured. T1DM development in rats induces a significant increase in SCr, BUN levels, and proteinuria. Interestingly, T1DM rats treated with liraglutide, metformin, or their combination have a significant decrease in BUN and SCr levels when compared with the untreated T1DM rats (Figure 1A–C). Functional changes of the kidney of the T1DM rats were associated with histopathological changes. PAS staining was performed on kidney tissues sections, and the results show that T1DM development in rats is associated with an increase in sclerosis and fibrosis as indicated by the black arrows in both the glomerular and tubular compartments of the diabetic kidneys. Intriguingly, treatment with liraglutide, metformin, or the combination significantly decreased sclerosis and fibrosis in the glomerular as well as the tubules observed in the kidneys of the T1DM rats (Figure 1D,E,H,I). Furthermore, the increase in collagen deposition observed in the glomerular and tubular compartments of the kidneys of the T1DM rats, as indicated by the black arrow, is significantly decreased upon treatment with liraglutide, metformin, or their combination (Figure 1F,G,J,K). Concomitantly, fibronectin mRNA levels increased in the kidney of the T1DM rats were significantly decreased upon treatment with liraglutide, metformin, or their combination (Figure 1L). Assessment of the slit diaphragm protein nephrin in T1DM rats shows a decrease in nephrin mRNA levels that was significantly restored, to almost control levels upon treatment with liraglutide, metformin or their combination (Figure 1M).

### 3.3. Treatment with Pharmacological Doses of Liraglutide, Metformin or Their Combination Reversed T1DM-Induced Alteration in Their Respective Signaling Targets

**Figure 2:**
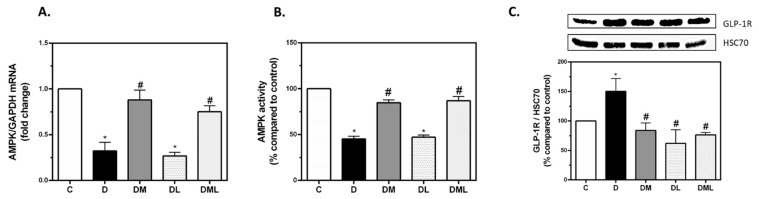
AMPK mRNA levels, AMPK activity assay and GLP-1R protein expression in kidney tissues. (**A**) Bar plot representing the differential expression of AMPK in all rat groups normalized against GAPDH expression. (**B**) Bar plot representing the differential AMPK activity in all rat groups. (**C**) Representative immunoblot of GLP-1R protein (up), and bar plot reflecting the quantification of GLP-1R protein expression (below) against HSC70. AMPK: adenosine monophosphate kinase, GAPDH: glyceraldehyde 3-phosphate dehydrogenase, GLP-1R: glucagon-like peptide-1 receptor, HSC70: heat shock cognate. All data are expressed as mean ± SEM. * *p* ≤ 0.05 diabetic vs. control groups. # *p* ≤ 0.05 treated vs. diabetic groups.

T1DM rats show a significant decrease in AMPK activity as underlined by the decrease in AMPK mRNA levels and the AMPK activity assay. Treatment with metformin or the combination of liraglutide and metformin significantly increased the levels of AMPK mRNA and AMPK activity when compared to the untreated diabetic group, whereas treatment with liraglutide had no significant effect on AMPK expression (Figure 2A,B). In parallel, T1DM rats had a significant increase in GLP-1R expression when compared to their control littermates. Treatment with liraglutide, metformin, or their combination significantly decreased GLP-1R protein expression when compared to the untreated T1DM group (Figure 2C), suggesting that AMPK could be a potential regulator of GLP-1R in diabetes.

### 3.4. DUOX1 and DUOX2 of the NADPH Family of Enzymes Play a Role in T1DM-Induced ROS Production

**Figure 3:**
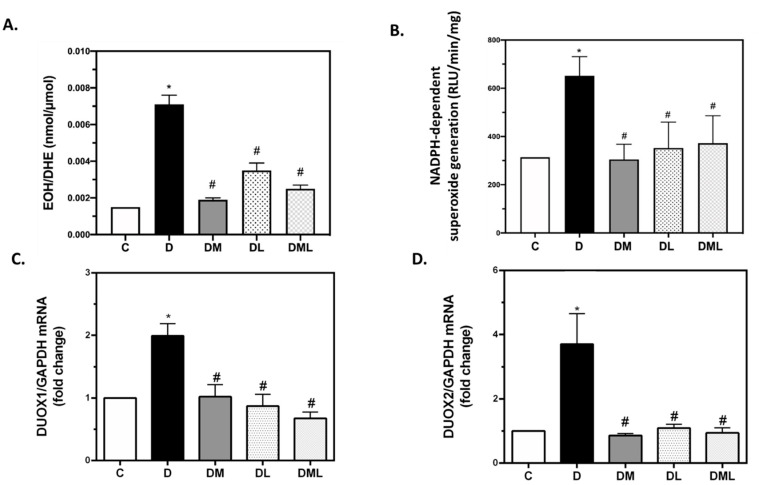
ROS production, NADPH oxidase activity and DUOX1/DUOX2 mRNA expression in the kidneys. (**A**) Bar plot reflecting ROS production through EOH/DHE ratio quantification. (**B**) Bar plot reflecting NADPH oxidase activity. (**C**,**D**) Bar plots representing the differential expression of DUOX1 and DUOX2 normalized against GAPDH expression respectively. GAPDH: glyceraldehyde 3-phosphate dehydrogenase. All data are expressed as mean ± SEM. * *p* ≤ 0.05 diabetic vs. control groups. # *p* ≤ 0.05 treated vs. diabetic groups.

As expected, ROS production in the T1DM group was increased when compared with their control littermate group. Interestingly, treatment of the T1DM rats with liraglutide, metformin, or their combination significantly decreased ROS production (Figure 3A). In parallel, and since NADPH oxidases are one of the major sources of ROS involved in T1DM-induced renal injury, we assessed NADPH oxidases activity in control, T1DM and T1DM treated rats with liraglutide, metformin or their combination. Our results show a significant increase in NADPH oxidases enzymatic activity in the T1DM group of rats, that was markedly dampened in all treated T1DM rats’ groups (Figure 3B). Importantly, and for the first time to our knowledge we show that DUOX1 and DUOX2 isoforms of the NADPH family of enzymes are regulated within T1DM development (Figure 3C,D). In fact, mRNA levels of both isoforms are increased in the T1DM group of rats. Of interest, treatment with liraglutide, metformin or their combination reduced T1DM-induced DUOX1 and DUOX2 activation as assessed by the decrease in their mRNA expression.

### 3.5. GLP-1R but Not AMPK Regulate TRPC6 Expression in T1DM Rats

**Figure 4:**
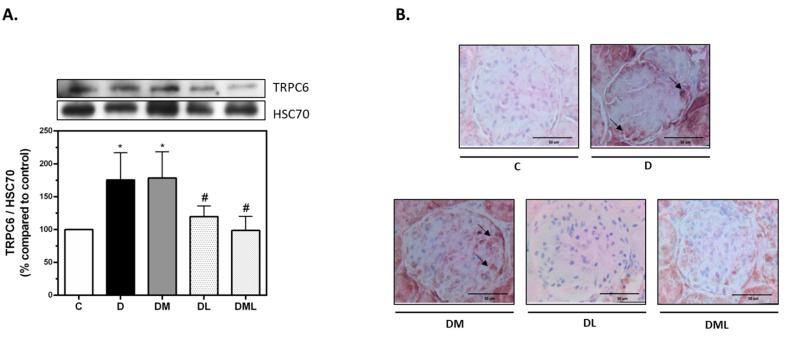
TRPC6 protein expression in the kidneys. (**A**) Representatives immunoblot of TRPC6 protein (up) and bar plot representing the quantification of TRPC6 expression against HSC70. (**B**) Representative microphotographies of the immunohistochemistry staining of TRPC6 as indicated by the black arrows (Magnification 40X, scale bar 50 µm). HSC-70: Heat shock cognate. All data are expressed as mean ± SEM. * *p* ≤ 0.05 diabetic vs. control groups. # *p* ≤ 0.05 treated vs. diabetic groups.

We screened for TRPC6 protein expression in the kidneys of the different groups of rats used in this study. Our results show a significant upregulation of TRPC6 in kidneys of the T1DM group when compared to their control littermate group. Of interest, T1DM-induced TRPC6 expression was attenuated upon liraglutide treatment or the combination of liraglutide and metformin treatment. However, TRPC6 expression was not affected when the T1DM rats were treated with metformin alone (Figure 4).

### 3.6. TRPC6 Interacts with DUOX1, DUOX2 and GLP-1R in Humans

**Figure 5:**
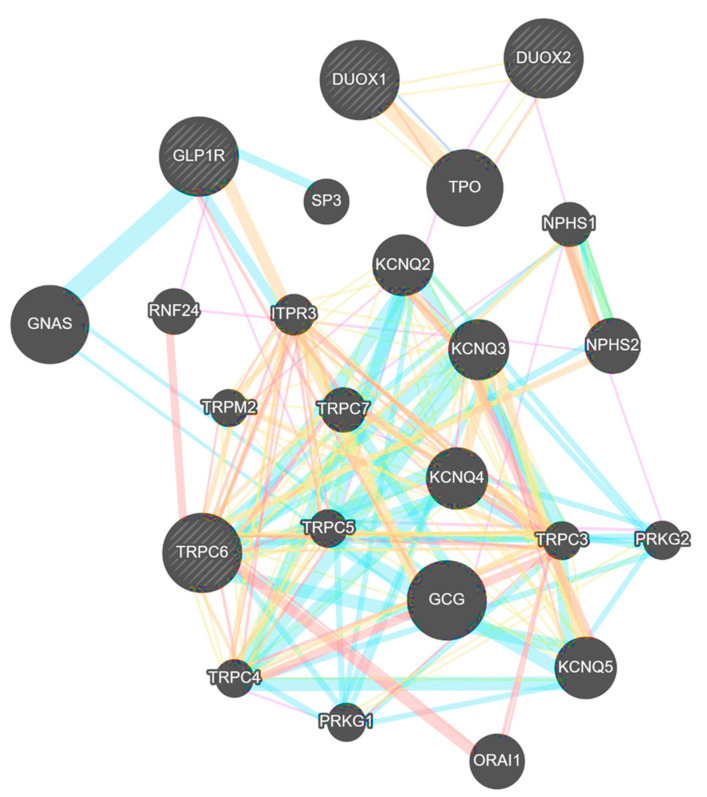
Protein interactions between TRPC6, DUOX1, DUOX2, GLP1-R, nephrin, podocin and other proteins in humans obtained from GeneMANIA database. Colors of the matching lines in the figure indicate different types of interactions: orange line indicates predicted interaction, blue line indicates shared pathway, green indicates genetic interactions, yellow indicates shared protein domains, pink indicates physical interactions, blue indicates co-localization and purple indicates co-expression. The width of the line reflects the significance of interaction: the thicker the line, the more robust the prediction of the interaction. TRPC: transient receptor potential channel, NPHS2: podocin, NPHS1: nephrin, RNF24: RING finger protein 24, KIRREL: Kin of IRRE-like protein 1, PLCG1: 1-phosphatidylinositol 4,5-bisphosphate phosphodiesterase gamma-1.

To associate our experimental animal model observations to clinical setting, GeneMANIA database were used to observe the crosstalk between GLP-1R, TRPC6 and DUOX1 and DUOX2. Our results show that TRPC6 interacts directly with other TRPC channels such as TRPC1, TRPC3, TRPC4, TRPC5 and TRPC7. Of interest, it interacts with DUOX1, DUOX2 and GLP-1R indirectly (Figure 5). These associations underscore the interconnectivity between the global regulation of calcium influx through the TRPC channels and oxidative stress. Although there is no data on a direct interaction between TRPC6 and nephrin or podocin, our analysis of several protein databases predict such interaction based on genetic interactions and shared pathways (https://www.pathwaycommons.org/pc/dbSnapshot.do?snapshot_id=7, accessed on 2 May 2021, https://pumed.ncbi.nlm.nih.gov/20482850/, accessed on 2 May 2021). Pathway enrichment using the gene ontology “GO” database showed that TRPC6 as well as other TRP channels are involved in calcium homeostasis and oxidative stress pathways and can be accountable for glomerular visceral epithelial cell development (Supplementary Table S2).

## 4. Discussion

**Figure 6:**
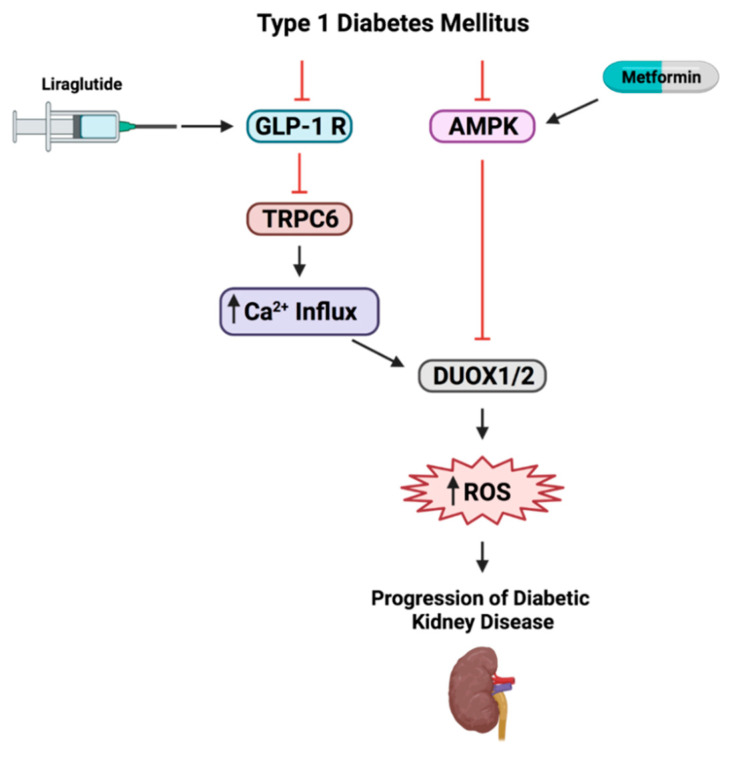
Graphical abstract. The reno-protective effect of liraglutide is mediated by the inhibition of TRPC6/NADPH oxidases-DUOX1 and 2-ROS production signaling pathways. However, metformin reno-protective effect is mediated by damping NADPH oxidases-DUOX1 and 2-ROS production axis through AMPK activation independently from TRPC6.

To our knowledge, this study is the first to show that in T1DM, the GLP1-RA, liraglutide, exerts its reno-protective effect by regulating TRPC6/NADPH oxidases-DUOX1 and 2 signaling axis. Herein, T1DM *Sprague–Dawley* male rats treated with liraglutide show a homeostatic adjustment in diabetes-induced increase in SCr, BUN and proteinuria associated with a reversal in fibronectin and collagen IV deposition and an increase in nephrin glomerular expression. Furthermore, the effect of liraglutide was mimicked by metformin and there was no synergistic effect when the combination of metformin or liraglutide were used. The observed functional, histopathological and molecular renal injuries in T1DM rats were associated with an increase in DUOX1 and 2-induced NADPH dependent ROS production and TRPC6 expression. Treatment with liraglutide restored the homeostatic signaling balance of DUOX1 and 2, decreased NADPH oxidases-induced ROS production and regulated TRPC6 expression. In contrast, treatment with metformin only attenuated the upregulation in ROS production resulting from the activation of the NADPH oxidases-DUOX1 and 2 without affecting the TRPC6 expression (Figure 6). Noteworthy, the observed reno-protective effect of liraglutide is independent of its glucose lowering action, as the increase in blood glucose levels and HbA1c % were maintained despite the treatment. These findings could pinpoint to new therapeutic strategies in the management of T1DM-induced DKD and could offer a tentative explanation about the debatable reno-protective effect of metformin in patients with type 1 diabetes.

In fact, the reno-protective effect of liraglutide is well documented in patients with type 2 diabetes mellitus (T2DM) [33], as liraglutide is one of the pharmacotherapeutic agents used to treat those patients. Interestingly, to our knowledge, our study reiterates on the protective effect of liraglutide, but herein in T1DM. In this study, treatment with liraglutide in T1DM *Sprague–Dawley* male rats reveals a reno-protective effect manifested by normalized SCr, BUN and proteinuria levels independently of blood glucose and HbA1C levels. Moreover, fibronectin and collagen IV are matrix proteins that may act as early markers for kidney fibrosis in diabetic nephropathy [34]. Treatment with liraglutide, diminish glycosylation and collagen deposition as assessed by PAS and MT staining and reduce fibronectin mRNA expression resulting in a decrease in renal fibrosis. Furthermore, the reduction in nephrin expression, a hallmark of diabetic podocyte injury [34], was restored upon treatment with liraglutide, thus indicating an amelioration of diabetic kidney injury. Furthermore, and to our knowledge this study is the first to highlight the mechanism behind the protective effect of liraglutide.

In contrast, besides its beneficial effect in patients with T2DM associated renal injury [35], it remains debatable whether metformin should be included as adjuvant therapy for patients with T1DM [17,36]. A plausible explanation to these contradictory clinical results when it comes to metformin, could be explained by the fact that metformin in type 1 diabetes cannot correct the alteration in the calcium influx known to stimulate respiratory chain activity leading to higher amounts of reactive oxygen species (ROS). However, in this study, our results with metformin or the combination of metformin and liraglutide revealed a reno- protective effect in the used animal model of type 1 diabetes. Hence, further studies should be performed to better delineate the mechanism of action of metformin to understand the contradiction observed in its reno-protective effect in the clinical trials on patients with T1DM.

Liraglutide increases intracellular cyclic AMP (cAMP) leading to insulin release in the presence of elevated glucose concentrations [37], while metformin lowers hepatic glucose production via mitochondrial uncoupling, diminishing oxidative stress and reducing leukocyte-endothelium interactions [38,39]. As a result, metformin enhances the insulin-mediated suppression of gluconeogenesis [40]. In this study, the animal model we are using is an STZ-induced type 1 diabetic model with no excretion of insulin (STZ induces β cells destruction). Therefore, it is reasonable that the glucose levels were not affected by the treatments.

A central mechanism for podocyte injury and diabetic nephropathy involves oxidative stress, particularly ROS production through NADPH oxidases [41]. As expected, ROS production is markedly elevated in untreated diabetic animals, and significantly reduced upon treatment with liraglutide and/or metformin. ROS production in the kidney is mainly due to the action of NOXs. Although NOX4 and NOX1 have been extensively described in renal tissues [42], to date, there is no data on renal DUOXs in DKD. The role of DUOX1 and 2 has been extensively studied in the context of thyroid cancer differentiation and progression [43], however, and to our knowledge this is the first study that describes their role in DKD. Our results show that T1DM is associated with an increase in both DUOX1 and DUOX2 mRNA levels concomitantly with an increase in NADPH oxidase activity and ROS production. However, treatment with liraglutide, metformin, or their combination reverses T1DM induced DUOX1 and 2 upregulation, NADPH oxidase activation and ROS production. In corroboration with these results, GLP-1 has been shown to reduce oxidative stress in T1DM and T2DM [44], but its effect on DUOX is still to be studied. Moreover, the anti-oxidative effects of the AMPK activator metformin were previously defined in DKD. In the present study, a cross-talk between AMPK and DUOX1 and 2 is described in T1DM induced DKD, however, the exact underlying mechanism still needs further elucidation. To note, that our group and others previously showed a link between AMPK signaling pathway and NOX4 in diabetic nephropathy [45].

DUOXs require calcium for its basal activation [7]. In this study, we identify a potential link between TRPC6 overexpression and the increased activity of NOX enzymes, specifically DUOX1 and DUOX2 and the resulting ROS production, leading to diabetic kidney injury. Of interest, ROS are important modulators of TRPC6 activation [46,47]; the simple addition of H2O2 can mobilize TRPC6 channels to the podocyte membrane and increase calcium influx. TRPC6 plays a major role in podocyte function and thus any disruption in its expression would affect podocyte structure and subsequent filtration process [47]. Our results show that in T1DM rats, TRPC6 expression is increased. This let to postulate that TRPC6 overexpression may lead to calcium influx increase, activating DUOX and ROS production. Furthermore, we show for the first time that liraglutide but not metformin reduces TRPC6 expression. These findings suggest that liraglutide can selectively inhibit TRPC6, thus, protecting the kidney through subsequent inhibition of ROS production. Beside the current study, little is known about the crosslink between GLP-1RA and TRP channels. Togashi et al. have shown that the knockdown of TRPM2 inhibited insulin release in response to the treatment with GLP-1RA [48].

To correlate our experimental data with clinical observations, the interaction among the different identified signaling molecules was assessed using the GeneMANIA gene interaction database. TRPC6 was found to be associated with other TRPC channels. These interactions reflect a crosstalk between TRPC channels in human tissue and subsequently the tight regulation of calcium influx through these channels. Furthermore, TRPC6 seems to interact directly with nephrin and podocin. These results suggest that the loss of nephrin observed could be strongly associated with calcium ion regulation through TRPC. Moreover, looking at the indirect interactions of TRPC6, the analysis revealed an association between DUOX1, DUOX2, TRPC6 and GLP-1R, a relation that has been confirmed in our T1DM rats experimental model.

## 5. Conclusions

In conclusion, our results show a promising role of liraglutide in delaying diabetic kidney injury and outline a dual action of liraglutide on TRPC6 and NADPH oxidases DUOX1 and 2. These findings could pinpoint new therapeutic strategies in the management of T1DM-induced DKD.

## Data Availability

The data that support the findings of this study are available from the corresponding author upon reasonable request.

## Supplementary Materials

The following are available online at https://www.mdpi.com/article/10.3390/biomedicines9101360/s1, Table S1: List of primers, Table S2: Gene Ontology biological process associated with TRPC6 protein interactions network obtained from GeneMANIA database.
