# Supplementary material for: Reno-Protective Effect of GLP-1 Receptor Agonists in Type1 Diabetes: Dual Action on TRPC6 and NADPH Oxidases
Source: Biomedicines. 2021 Sep 30;9(10):1360. doi: 10.3390/biomedicines9101360 (PMC8533165; doi:10.3390/biomedicines9101360)
Supplement: Supplementary file 1 [file biomedicines-09-01360-s001.zip › biomedicines-1318169-supplementary.pdf]

Table S1. List of primers.

Primer	Sequence
Fibronectin (rFn)	F: 5′-CGGGAACATCATCGGATCGT-3′<br>R: 5′-GGAGAACCAGGAGAGCACAC-3′
Nephrin (rNph)	F: 5′-GTGACCTCAGTGATGACGCA-3′<br>R: 5′-TAGGAGACACAAGCTCGGGA-3′
DUOX1 (DUOX-1)	F: 5′-AGGCACTGGTGGAAAACATC-3′<br>R: 5′-GGAGAAAAGGTGCCTGAAAA-3′
DUOX2 (DUOX-2)	F: 5′-GAAGTCCACAGCAGCATCAA-3′<br>R: 5′-CCACGGACATTGAAGAAACC-3′
AMPK (AMPK-a1)	F: 5′-GCAGTTGCCTACCACCTCAT-3′<br>R: 5′-GTACGCCTTGGTGTTTGGAT-3′
GAPDH	F: 5′-GGGGCTCTCTGCTCCTCCTG-3′<br>R: 5′-CGGCCAAATCCGTTCACACCG-3′

Table S2. Gene Ontology biological process associated with TRPC6 protein interactions network obtained from GeneMANIA database.

Gene	Description	Rank
DUOX1	dual oxidase 1 [Source:HGNC Symbol;Acc:HGNC:3062]	N/A
DUOX2	dual oxidase 2 [Source:HGNC Symbol;Acc:HGNC:13273]	N/A
GLP1R	glucagon like peptide 1 receptor [Source:HGNC Symbol;Acc:HGNC:4324]	N/A
TRPC6	transient receptor potential cation channel subfamily C member 6 [Source: HGNC Symbol;Acc:HGNC:12338]	N/A
GCG	glucagon [Source:HGNC Symbol;Acc:HGNC:4191]	1
GNAS	GNAS complex locus [Source:HGNC Symbol;Acc:HGNC:4392]	2
TPO	thyroid peroxidase [Source:HGNC Symbol;Acc:HGNC:12015]	3
KCNQ5	potassium voltage-gated channel subfamily Q member 5 [Source:HGNC Symbol;Acc:HGNC:6299]	4
KCNQ4	potassium voltage-gated channel subfamily Q member 4 [Source:HGNC Symbol;Acc:HGNC:6298]	5
KCNQ2	potassium voltage-gated channel subfamily Q member 2 [Source:HGNC Symbol;Acc:HGNC:6296]	6
KCNQ3	potassium voltage-gated channel subfamily Q member 3 [Source:HGNC Symbol;Acc:HGNC:6297]	7
NPHS2	NPHS2 podocin [Source:HGNC Symbol;Acc:HGNC:13394]	8
ORAI1	ORAI calcium release-activated calcium modulator 1 [Source:HGNC Symbol; Acc:HGNC:25896]	9
RNF24	ring finger protein 24 [Source:HGNC Symbol;Acc:HGNC:13779]	10
SP3	Sp3 transcription factor [Source:HGNC Symbol;Acc:HGNC:11208]	11
TRPC7	transient receptor potential cation channel subfamily C member 7 [Source: HGNC Symbol;Acc:HGNC:20754]	12
NPHS1	NPHS1 nephrin [Source:HGNC Symbol;Acc:HGNC:7908]	13
ITPR3	inositol 1,4,5-trisphosphate receptor type 3 [Source:HGNC Symbol;Acc: HGNC:6182]	14
PRKG2	protein kinase, cGMP-dependent, type II [Source:HGNC Symbol;Acc:HGNC: 9416]	15
PRKG1	protein kinase, cGMP-dependent, type I [Source:HGNC Symbol;Acc:HGNC: 9414]	16
TRPC3	transient receptor potential cation channel subfamily C member 3 [Source: HGNC Symbol;Acc:HGNC:12335]	17
TRPC4	transient receptor potential cation channel subfamily C member 4 [Source: HGNC Symbol;Acc:HGNC:12336]	18
TRPC5	transient receptor potential cation channel subfamily C member 5 [Source: HGNC Symbol;Acc:HGNC:12337]	19
TRPM2	transient receptor potential cation channel subfamily M member 2 [Source: HGNC Symbol;Acc:HGNC:12339]	20
